# Genetic Evaluation of Inpatient Neonatal and Infantile Congenital Heart Defects: New Findings and Review of the Literature

**DOI:** 10.3390/genes12081244

**Published:** 2021-08-14

**Authors:** Benjamin M. Helm, Benjamin J. Landis, Stephanie M. Ware

**Affiliations:** 1 Department of Medical and Molecular Genetics, Indiana University School of Medicine, Indianapolis, IN 46202, USA; stware@iu.edu; 2 Department of Epidemiology, Indiana University Fairbanks School of Public Health, Indianapolis, IN 46202, USA; 3 Department of Pediatrics, Indiana University School of Medicine, Indianapolis, IN 46202, USA; benjland@iu.edu

**Keywords:** congenital heart disease, chromosome microarray analysis, genetic testing, mutation, dysmorphic, cardiac classification, geneticist, exome

## Abstract

The use of clinical genetics evaluations and testing for infants with congenital heart defects (CHDs) is subject to practice variation. This single-institution cross-sectional study of all inpatient infants with severe CHDs evaluated 440 patients using a cardiovascular genetics service (2014–2019). In total, 376 (85.5%) had chromosome microarray (CMA), of which 55 (14.6%) were diagnostic in syndromic (N = 35) or isolated (N = 20) presentations. Genetic diagnoses were made in all CHD classes. Diagnostic yield was higher in syndromic appearing infants, but geneticists’ dysmorphology exams lacked complete sensitivity and 6.5% of isolated CHD cases had diagnostic CMA. Interestingly, diagnostic results (15.8%) in left ventricular outflow tract obstruction (LVOTO) defects occurred most often in patients with isolated CHD. Geneticists’ evaluations were particularly important for second-tier molecular testing (10.5% test-specific yield), bringing the overall genetic testing yield to 17%. We assess these results in the context of previous studies. Cumulative evidence provides a rationale for comprehensive, standardized genetic evaluation in infants with severe CHDs regardless of lesion or extracardiac anomalies because genetic diagnoses that impact care are easily missed. These findings support routine CMA testing in infants with severe CHDs and underscore the importance of copy-number analysis with newer testing strategies such as exome and genome sequencing.

## 1. Introduction

Congenital heart defects (CHDs) are the most prevalent type of birth defect, with an estimated global prevalence of ~1–2% [1,2]. CHD etiologies are diverse, and it is currently estimated that up to 20–30% of cases have an identifiable genetic or environmental etiology [3]. It is expected that the proportion of CHDs with an identifiable genetic cause will increase with the ongoing expansion of genomic testing. Single-gene disorders are identified in 3–5% of cases, chromosome aneuploidy is identified in 8–10% of cases, and chromosome copy-number variants (CNVs) are identified in 3–25% of cases [2,3]. The remaining 60–80% of cases may be caused by novel genetic and epigenetic risk factors awaiting identification. Novel applications of exome and whole-genome sequencing (ES/WGS) are showing diagnostic yields ranging approximately 5–30% for CHDs. However, there is considerable variability in the methodology of CHD ES/WGS studies to date [4,5,6,7,8,9,10].

Chromosomal aneuploidy and CNVs are detectable by chromosome microarray analysis (CMA). Previous studies have demonstrated a CMA diagnostic yield of 3–10% and 3–25% for isolated CHDs and syndromic CHDs, respectively, and CMA has been discussed as a first-tier diagnostic tool for investigating CHD causes [2,11,12]. Previous literature recommended routine consideration of cytogenetic testing based on CHD lesion type, e.g., fluorescence in situ hybridization (FISH) for conotruncal defects in 22q11.2 deletion syndrome and supravalvular aortic stenosis in Williams syndrome [13,14,15]. However, clinical genetic testing practices for CHDs vary significantly across institutions, and similarly, CMA may be inconsistently utilized as a first-tier test [16]. In addition, there have been limited studies of CMA investigations in clinical CHD cohorts, though more recent studies report overall diagnostic CMA yields of up to 14–24% depending on whether CHD is thought to be isolated or is seen in conjunction with dysmorphic features or extracardiac anomalies (ECAs) [17,18,19,20]. Differences in cohort ascertainment, type of genetic testing used, documentation of dysmorphology or other phenotypic features, and age of patient testing or evaluation make comparisons difficult. Reports of diagnostic yield and relevant findings from CMA and other forms of genetic testing in clinical CHD programs that have adopted systematic testing approaches are needed to limit bias. Genetic evaluation is an important part of the care of pediatric and adult CHD patients alike, helping to confirm diagnoses, inform medical management, and improve genetic counseling [2]. Findings from CMA screening in CHD populations should help to (1) provide evidence-based data to guide the use of CMA/CNV detection in CHD patients to achieve early genetic diagnoses and (2) improve knowledge of genetic causes of CHDs identifiable by both CMA and ES/WGS in the future.

Starting in 2014, our center established a cardiovascular genetics inpatient service for infants with CHDs. The program deployed an algorithm to standardize clinical genetics assessment of CHD patients, including routine genetic testing largely agnostic to the lesion type but informed by input from clinical genetics providers. This inpatient service sought to ensure that an evaluation by a medical geneticist occurred in neonates and infants admitted for care due to critical CHD; CMA testing was standard except in patients with aneuploidy, though other forms of genetic testing may have been completed in place of, or in addition to, CMA. The goal of this study was to assess the testing results from the inpatient service as defined by diagnostic yields of CMA testing standardized as a first-tier test for most CHD patients, with a specific focus on clinically significant results. A second goal was to explore the proportion of molecular genetic diagnoses not detectable by CMA. In either case, we sought to determine the yield of genetic testing in syndromic and apparently isolated CHDs, with a long-term goal of determining CHD subpopulations that would benefit most from genetics evaluation. In our study, we retrospectively reviewed CMA and molecular genetic testing findings for 440 patients evaluated in our program from 2014 through 2019. We assess these results in the context of previous studies and evolving genetic testing options. This work contributes to knowledge about CNV causes for CHDs in addition to informing standardization of clinical genetic testing practices for CHD populations in pediatric inpatient settings.

## 2. Materials and Methods 

This single-institution descriptive study involved review of deidentified aggregate clinical data in neonates and infants (≤1 year of age) with CHDs prospectively assessed by the inpatient cardiovascular genetics service. The Indiana University Institutional Review Board (IRB protocol #2004409740) deemed this study exempt after review.

### 2.1. Study Population and Inclusion Criteria

The study population consisted of neonates and infants with CHDs in the cardiovascular and neonatal intensive care settings referred for cardiovascular genetics evaluation at Riley Hospital for Children (Indiana University Health) from August 2014 through December 2019. No patients were excluded from review. The primary data collected included the following: CHD lesion class using the Botto classification [1], presence of ECAs, completion of CMA, and CMA results (normal, abnormal but of uncertain significance, and diagnostic/abnormal with clinical significance). Non-CMA genetic testing results were also reviewed to assess the diversity of genetic tests ordered and/or redundant testing completed. All CMAs included were performed either within our institution or by the referring hospital system when copies of original results were obtained and verified. Since this study focused on diagnostic findings from genetic testing, we do not report on diagnoses based solely on clinical examination in this cohort, e.g., VACTERL association or Goldenhar syndrome.

### 2.2. Clinical Algorithm for Inpatient Cardiovascular Genetics Evaluations

An algorithm was deployed to optimize referral of patients for genetics assessment largely based on that of Cowan and Ware [3]. Generally, any CHD infant requiring intensive care hospitalization was recommended for genetics evaluation, and at minimum, CMA testing was recommended. However, patients with isolated septal defects were not routinely referred unless the following criteria were met: (1) there was a family history of CHDs or (2) there were dysmorphic features or ECAs suspicious for an underlying syndrome. Likewise, patients with common aneuploidies were not a focus of the program, but the inpatient service was consulted on a smaller number that presented in an atypical fashion. Otherwise, CMA was recommended for all CHD classes unless other forms of genetic testing supplanted it based on medical genetics provider input. For example, a patient may have had striking features highly specific for a group of syndromes for which molecular genetic testing was recommended over CMA (e.g., Noonan syndrome/RASopathies) or non-CMA cytogenetic testing such as FISH or karyotype was recommended for a priori suspicion for a specific disorder (e.g., trisomy 21/18/13). In cases where additional testing beyond CMA was recommended, a two-step strategy involving reflex to molecular genetic testing after the initial CMA was normal was the most common approach (including ES, phenotype-specific gene panels, etc.) following CMA. In most cases, reflex molecular genetic testing following CMA was utilized for cases with possible syndromic presentations.

### 2.3. CHD Classification

Patients’ CHDs were classified into mutually exclusive CHD categories based on the Botto classification scheme, specifically Level 3 classes including the following: anomalous pulmonary venous return (APVR); atrioventricular septal defects (AVSD); complex, conotruncal, heterotaxy/laterality spectrum defects; left ventricular outflow tract obstructions (LVOTO); right ventricular outflow tract obstructions (RVOTO); and septal defects [1]. A pediatric cardiologist with CHD classification expertise (BJL) adjudicated questionable cases. CHDs were classified using the “complex” class when multiclass constellations of CHDs were identified that could not be certainly categorized into one Level 3 class.

### 2.4. Clinical Evaluations and Defining Apparently Isolated vs. Syndromic CHDs

All patients had evaluations by a board-certified medical geneticist on call for inpatient consults, including documentation of dysmorphology examinations, presence or absence of ECAs, and genetic testing strategies. Patients received pre- and post-test genetic counseling as appropriate. For this study, ECAs were defined as (1) presence of any noncardiac organ malformation(s) and/or major anomalies and/or (2) any constellation of anatomical dysmorphisms not considered normal population variation and deemed minor anomalies. These were determined by medical geneticists who followed guidelines for defining minor vs. major anomalies in the literature [21,22]. Other clinically significant medical issues indicative of a possible syndrome such as hypo- or hypercalcemia, immunodeficiency, or seizures were noted. Due to the age of this cohort, we did not typically assess development. Clinical examination and additional testing or imaging as appropriate verified the presence of ECAs. When CHDs were accompanied by one or more features in either of the categories above, cases were considered syndromic in presentation. Presentations without ECAs or significant dysmorphisms were classified as apparently isolated CHDs.

### 2.5. Genetic Testing and Results Classification

CMAs were performed in clinical laboratories using standard methods. In-house CMA was performed on genomic DNA extracted from peripheral blood using the Applied Biosystems CytoScan HD array platform (ThermoFisher Scientific, Carlsbad, CA, USA) consisting of 1,953,246 unique non-polymorphic copy-number probes and 743,304 single nucleotide polymorphism probes spanning the whole genome. The CNVs were analyzed and reported using the NCBI human genome build 37.1 (GRCh37/hg19) by board-certified cytogeneticists. When CMA was completed at outside facilities, these methods were generally the same, as verified by review of the original test reports. Geneticists and genetic counselors reviewed CMA reports, including clinical interpretation of CNVs, as part of standard inpatient consultation practices. For this study, CMA abnormalities and molecular genetic testing results were broadly classified as (1) normal, (2) variants of uncertain significance (VUSs), or (3) diagnostic and clinically significant (i.e., pathogenic or likely pathogenic results). The last two categories were verified by combined review by laboratory geneticists/cytogeneticists and clinical genetics teams using standard variant interpretation practices. Clinically significant CMA results were defined by pathogenic and likely pathogenic results confirming a genetic/syndromic diagnosis, unequivocally explaining the cardiac phenotype, informing risk counseling, altering medical management, and/or identifying a clinically relevant secondary finding. Strict interpretation was used for all VUS results, so no candidate CNVs or molecular VUSs were considered in the proportion of clinically significant or diagnostic results in this study due to limited evidence. Molecular genetic testing, including exome sequencing (singleton, duo, or trio samples as able) and phenotype-targeted next-generation sequencing gene panels, was performed by commercial CAP/CLIA-approved genetic testing laboratories in the United States using standard methodologies. Genomic coordinates and/or specified breakpoints for chromosome CNVs can be requested from the corresponding authors.

### 2.6. Descriptive Statistics and Analyses

We performed post hoc review of aggregate data to determine the proportion of clinically significant CMA/genetic testing findings that were associated either with presence/absence of ECAs or prior suspected genetic diagnosis based on the clinical examination. Aggregate data analysis and descriptive statistics were calculated, and categorical analyses and comparison of proportions were performed using chi-squared tests of association or Fisher’s exact tests for small samples. Inferential statistical testing *p*-values used a threshold of α < 0.05 for statistical significance. Analyses were performed in SAS version 9.4 (SAS Institute, Cary, NC, USA).

## 3. Results

### 3.1. Overview of Cohort

From 2014 through 2019, the cardiovascular genetics service evaluated a total of 440 unique CHD infants (56% male, *n* = 246/440), representing all CHD classes (Table 1). More males (56%) were represented in this cohort compared to females (Z = 3.5, *p* = 0.0004). The three most common CHD classes represented in this cohort were conotruncal (130/440, 29.6%), LVOTO (121/440, 27.5%), and complex (66/440, 15.0%). Proportions of CHD classes and male vs. female sex are similar to previous studies of inpatient CHD cases that also followed Botto classification [18]. Overall, 30% (*n* = 132/440) of patients were noted to have ECAs, and ECA status was associated with CHD class (χ^2^ = 105.5; *p* < 0.0001). ECAs were expectedly most common in the heterotaxy/laterality disorders group (ECAs present in 32/35, 91.4%) compared to all other CHD classes. The three heterotaxy cases without ECAs were laterality spectrum cardiovascular malformations such as dextrocardia. ECAs were also prevalent in the septal CHD class (15/24, 62.5% of septal cases with ECAs), as was expected based on the clinical algorithm. These may represent patients with more severe ECAs requiring intensive care despite “simpler” CHDs. ECAs were relatively less common in the APVR, AVSD, and complex classes. When excluding heterotaxy, an association remained between CHD class and presence of ECAs (χ^2^ = 42.0; *p* < 0.0001). Overall, our program sought to standardize ordering CMA across all Botto classes, and statistical analysis indicates no differences in the proportions of CMAs ordered across classes (χ^2^ = 3.1; *p* = 0.88), suggesting no bias in genetic testing based on CHD class. 

### 3.2. Overview of Genetic Testing Practices

**Figure 1:**
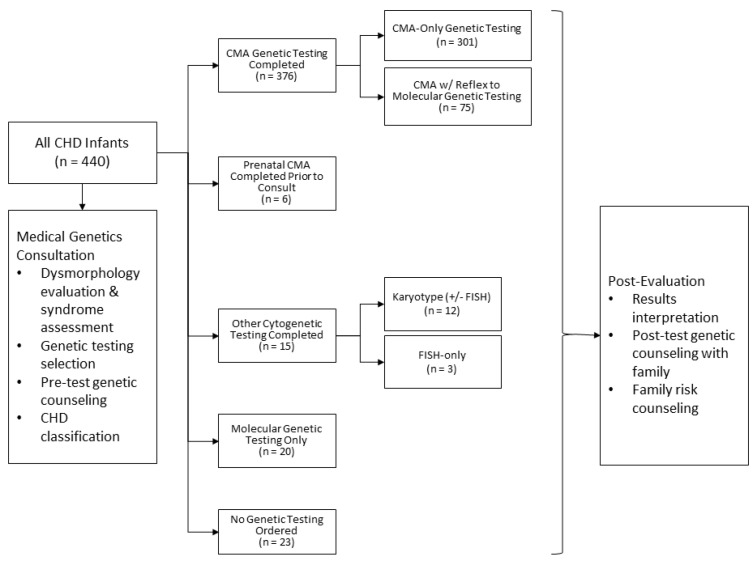
Genetic testing modalities used in evaluation of CHD inpatient cohort.

Figure 1 summarizes the genetic testing documented in this study. In this cohort, 376/440 (85.5%) patients had CMA testing completed. The remaining 64/440 (14.5%) patients did not have CMA testing for a variety of reasons. These included 20 that had molecular genetic testing prioritized over CMA (20/64, 31.3%), 12 that had karyotype plus FISH only without reflex to CMA (18.8%), 3 that had FISH only (4.7%), 6 cases that had prenatal CMA completed, and 23 that had no genetic testing ordered (35.9%). Of the group that had no genetic testing completed, 11/23 (47.8%) were due to inpatient ordering error after the geneticist recommended testing, and 10/23 (43.5%) were due to geneticists deferring testing based on exam. The remaining two cases without genetic testing had previous cytogenetic testing completed at outside hospitals, and records were obtained for review.

### 3.3. Diagnostic Yield of CMA

Of the 376 CMAs completed by our service, the overall diagnostic yield of clinically significant abnormal results was 14.6% (*n* = 55/376). Table 2 summarizes the proportions of completed CMAs per Botto class, and the results are sectioned by CMA results that were abnormal (includes variants of uncertain significance (VUSs)) and those that were considered abnormal and clinically significant (i.e., diagnostic for disease or genetic risk). While there were 56 cases of diagnostic CMA results, one was a heterotaxy case that had a deletion involving the *HBB* gene consistent with carrier status of β-thalassemia. While this provided information relevant for reproductive risk counseling, it was ultimately not counted towards the final total of diagnostic CMA results (*n* = 55). Otherwise, 264/376 (70.2%) CMA results were normal, and finally, 56/376 (14.9%) of the abnormal CMA results were considered VUSs. These latter results may reflect findings warranting further research investigation.

Table 3 summarizes CHD presentation and diagnostic yield of CMA in ECAs and apparently isolated CHD groups in the overall cohort (n = 440). As expected, the diagnostic yield of CMA testing is higher in syndromic patients than in those with apparently isolated CHDs. Of note, however, diagnoses were made by CMA in this latter group even though all patients had an evaluation by a medical geneticist who documented no significant dysmorphology. On review of the specific abnormalities, many were CNVs that have been associated with CHDs and neurodevelopmental features with highly variable phenotypes such as CNVs involving 16p11.2, 16p13.11, or 8p23.1 duplication.

### 3.4. Geneticists’ a Priori Assessment of Likelihood of Genetic Testing Abnormalities

Post hoc review of the clinically significant CMA results highlighted a diversity of cytogenetic diagnoses. The clinical algorithm called for geneticist consultation for all infants in the intensive care units with critical CHDs except in cases where a diagnosis of aneuploidy had been made prenatally or clinically at delivery. This provided an opportunity for retrospective evaluation of the medical geneticists’ differential and assessment of the likelihood of a clinically significant genetic abnormality being identified in the patient. We reviewed the consults to determine whether the medical geneticist assessed a low or high likelihood of a genetic syndrome or genetic finding a priori based on exam of the CHD neonates and infants (Table 4). Of the 55 cases with clinically significant CMA results, 63.6% (35/55) occurred in patients assessed as having a high likelihood of having a genetic abnormality. The remaining 36.7% (20/55) occurred in cases that were assessed as having a low likelihood of a genetic abnormality. Table 4 summarizes all clinically significant CMA findings.

Overall, the number of cases in each class is small, which limited statistical analyses; however, patterns emerged for some classes. For example, the conotruncal class had more clinically significant CMA findings, driven by the increased prevalence of 22q11.2 deletion syndrome and the presence of ECAs and/or dysmorphic features. Nineteen of twenty-two conotruncal cases (86.4%) with diagnostic CMA were deemed to have a high likelihood of a genetic abnormality based on the pre-CMA clinical evaluation, and 3/22 apparently isolated conotruncal cases had clinically significant CMA results (including 22q11.2 deletion syndrome). Similarly, the septal class had four cases with clinically significant CMA results, all occurring in patients in whom initial evaluation by a medical geneticist raised concern for high likelihood of a genetic syndrome. Interestingly, for the LVOTO class, there were more significant CMA results in patients with apparently isolated CHDs or whose pre-CMA clinical evaluations were not suspicious for syndromic presentations. Ten of sixteen (62.5%) of the clinically significant CMA results occurred in those with apparently isolated CHDs. Additional findings of interest within specific CHD classes are given below. Relevant ECAs and dysmorphic features documented by the consulting geneticists can be found in Supplementary Tables S1 and S2.

#### 3.4.1. APVR

Only one patient had a clinically significant result, a 15q11.2 deletion (BP1-BP2) that has been associated with risk of neurodevelopmental and learning disorders in addition to CHDs [23]. Similar 15q11.2 CNVs were identified in other CHD classes, and there are emerging data suggesting a possible role for these CNVs with CHDs [24]. Otherwise, no other APVR cases had clinically significant CMA findings. The patient with APVR was not suspected of having a genetic syndromic diagnosis at the time of geneticist evaluation.

#### 3.4.2. AVSD

For the AVSD class, two cases had clinically significant CMA diagnoses, and both had presence of significant ECAs: one patient had trisomy 21 and another had a large 10 Mb duplication 5p13.2-p11. The trisomy 21 case was diagnosed clinically by the geneticist and had concurrent karyotype and CMA indicating genetic testing redundancy.

#### 3.4.3. Complex

For the complex CHD class, 6/55 (10.9%) had significant results, including two whom the geneticist evaluation identified as low risk. Interesting diagnoses identified as high risk for a genetic abnormality included recombinant chromosome 8 syndrome, 22q11.2 duplication, and a large 2q22.1–q23.3 deletion encompassing the *ZEB2* gene consistent with Mowat–Wilson syndrome.

#### 3.4.4. Conotruncal

As described above, the conotruncal class had one of the highest CMA yields, with 22/115 (19.1%) having significant results. The most prevalent diagnosis in this class included 22q11.2 deletion syndrome, which occurred in 59.1% (*n* = 13/22) of these cases, though there was a variety of other diagnoses in the remaining 40.9%. Other recurrent diagnoses in the conotruncal group included 16p11.2 deletion syndrome (*n* = 2/22), both of whom were noted to have dysmorphic features and/or ECAs. More detailed cardiac phenotype information specifically for the conotruncal class cases is available in Supplementary Table S3a. Notably, the most prevalent subtypes of conotruncal lesions represented in this study were tetralogy of Fallot, dextro-transposition of the great arteries, and more uncommonly, double-outlet right ventricle and interrupted aortic arch. Supplementary Table S3b describes the various conotruncal lesion subtypes represented in cytogenetic diagnoses made in this study. Due to small sample sizes at the conotruncal subtype level, statistical analysis was not performed.

#### 3.4.5. Heterotaxy

For the heterotaxy class, 1/30 (3.3%) cases had a clinically significant result, a 16p13.11 duplication involving the *MYH11* gene associated with risk of aortic aneurysm [25], although this finding does not explain the heterotaxy phenotype. There was one other heterotaxy case that had an 11p15.4 deletion involving the *HBB* gene cluster associated with β-thalassemia carrier status and familial reproductive risk. This latter case was not included in the overall diagnostic yield, but it was considered a clinically significant secondary finding for familial risk counseling. Previous studies have indicated a CMA diagnostic rate of 10–20% in heterotaxy patients [3,12,26,27], but this was not seen in our cohort. Given the fact that other causes of heterotaxy such as variants in *ZIC3* or in genes causing primary ciliary dyskinesia or other ciliopathies require molecular testing to identify, many of these patients had additional molecular testing. The algorithm in place from 2014–2019 did not include molecular genetic testing, which was considered by consulting geneticists as clinically indicated and was not standardly applied to heterotaxy cases.

#### 3.4.6. LVOTO

For the LVOTO class, 16/101 (15.8%) had clinically significant results, including a diversity of cytogenetic diagnoses. While there was no clear enrichment for specific diagnoses like the association seen in conotruncal defects, there appeared to be more cases of apparently isolated LVOTO defects with clinically significant CMA results. This suggests that even for apparently isolated, nonsyndromic LVOTO cases, there should be consideration for ordering CMA given the cytogenetic diagnoses found here. Overall, for the LVOTO class, there were recurring CNVs in the 8p23.1, 15q11.2 (BP1-BP2), 16p11.2, 17p12, and 22q11.2 regions (Table 4). There was one LVOTO case with a 7q11.23 deletion consistent with Williams syndrome.

#### 3.4.7. RVOTO

For the RVOTO class, 3/32 (9.4%) had clinically significant results. The CNVs at 8p23.1 and 16p11.2 have not had extensive characterization with regard to the class of CHD in which they are most common. Here we note that the 8p23.1 deletion is present in the RVOTO class, whereas two 8p23.1 duplications were found in the LVOTO class. In contrast, both LVOTO and RVOTO classes have patients with 16p11.2 duplication. Tetrasomy X is a rare condition in which individuals may have very subtle dysmorphic features. CHD is associated with Tetrasomy X, with PDA and VSDs reported previously, but specific details of CHDs in all patients are lacking in the literature.

#### 3.4.8. Septal

The septal class had the highest CMA yield along with the AVSD class. There were 4/20 (20%) having significant results; however, all of the patients here were notable for presence of ECAs with pretest suspicion for syndromic etiologies. There were recurring diagnoses of 22q11.2 deletion syndrome. A second case of Mowat–Wilson syndrome caused by a large 2q22.1-q22.3 deletion encompassing the *ZEB2* gene was identified in this population. The pattern of the septal class having one of the higher CMA diagnostic yields may reflect a clinical algorithm biased toward syndromic septal defects instead of apparently isolated cases in the inpatient setting. It is also possible that infants with septal defects and underlying genetic syndromes may be more likely to require intensive care due to more severe clinical presentations. This warrants further investigation and comparison to other centers’ experiences.

### 3.5. Results of Molecular Testing

In 20/440 (4.5%) patients, molecular genetic testing was favored over standard CMA recommendations. Testing was diagnostic in 1 of these 20 patients. Fifteen (75%) of these occurred later in the study timeframe during the transition to utilizing next-generation sequencing panel testing for congenital anomalies and CHDs but also provided CNV information (all were nondiagnostic). Other molecular testing performed preferentially over CMA included two patients with first-tier heterotaxy gene panels and two patients with aortopathy panels. One of the aortopathy panels was ordered for the indication of familial supravalvular aortic stenosis since the panel includes the causative *ELN* gene. These tests were nondiagnostic. However, there was one exome sequencing with mtDNA sequencing test that was diagnostic, identifying a pathogenic mitochondrial variant in the *MTTL1* gene (m.3243A>G) that likely explained a cardiomyopathic phenotype but not necessarily the conotruncal anomaly in the patient. Therefore, for patients who had molecular genetic testing only (and no CMA), 1/20 (5%) had diagnostic results (overall cohort, 1/440 (0.2%)). All other results were either normal or involved VUS findings.

In 75/440 (17%) patients, molecular genetic testing was performed in addition to CMA due to a high likelihood of a genetic diagnosis. A total of 81 molecular tests were ordered in these 75 patients. Nine had molecular diagnoses that would have been missed by CMA alone (9/75, 12%), leading to an overall cohort proportion of 9/440 (2%) having molecular diagnoses. Table 5 provides a summary of the testing and overall yield for the 95 individuals (101 molecular tests) which includes the 75 individuals with, and 20 patients without, CNV results. With 10 positive tests in 95 individuals, the diagnostic yield of molecular testing in those individuals who underwent it was 10.5%, and the yield in the entire cohort, most of whom did not have molecular testing as neonates, was 2.3% (10/440). The highest yield was seen in CHARGE syndrome, Noonan/RASopathies, and Alagille syndrome. Each of these syndromes has well-defined dysmorphology and extracardiac features that can sometimes be seen in infancy, allowing the differential to be narrowed. We note that some of the patients with negative testing for CHARGE or Noonan could be given a clinical diagnosis in the future if they fulfilled clinical criteria since molecular testing does not diagnose every individual with these syndromes. The range of panels ordered demonstrates the variability of presentations of infants with CHDs.

## 4. Discussion

Clinical genetics evaluation and genetic testing have been recommended to inform diagnosis, management, and genetic counseling for CHD patients [2]. However, these recommendations are inconsistently applied, and genetic testing practices for CHD patients are similarly inconsistent across institutions [16]. Despite recognition of the genetic basis of CHD and recommendations for genetic testing, guidance about referring patients for evaluation by a medical geneticist has been less clear. Decisions about which CHD patients to refer, when to test, what modality to use, and when genetic testing and evaluation should be performed are all subject to practice variation. This creates the problem of the “missing denominator” in studies: CHD patients who are not thought to have high likelihood of a genetic syndrome based on their cardiac or other features do not undergo genetic evaluation or testing. Additional barriers to standardization have included continually evolving genetic testing technology, decreased access to geneticist expertise in some centers, and variable training in cardiovascular genetics among geneticists and cardiologists. In this study, we report genetic results in neonates and infants with CHDs after implementing a clinical algorithm to comprehensively evaluate and test CHD patients, prioritizing CMA as a first-tier diagnostic test. By incorporating an evaluation by a medical geneticist for each case, syndromic CHD phenotypes were clearly delineated from isolated CHDs. We demonstrate that all classes of CHD have diagnostic genetic findings and determine the yield for first-tier CMA testing. We find that evaluation by a medical geneticist correctly identifies many CHD patients with diagnostic findings as syndromic but misses a subset of patients with CHDs without significant dysmorphology or ECA. In Table 6, we compare findings of clinical genetic testing in CHD patients from published retrospective studies. Recent prenatal testing results are included for comparison.

The estimated yield of CMA for CHD patients has ranged approximately 3–25% based on prior studies.

Comparison with prior studies demonstrates the wide variability in study designs and cohort ascertainment that make direct comparisons of diagnostic yield difficult. Despite this, some important conclusions can be drawn as detailed below.

### 4.1. CHD Type Influences Diagnostic Yield

Several studies, including prenatal studies, have previously demonstrated an increased diagnostic yield in the septal or atrioventricular canal classes of CHD [18,20,28,37,38] despite differing in their inclusion and exclusion criteria. In the current study, the highest yield occurred for septal and AVSD defects (20%), and this may either reflect characteristics of septal patients in the acute cardiac care setting (likely enriched for more medically complex septal cases) and/or the clinical algorithm that prioritized septal patients that had a priori suspicion for genetic diagnoses. Notably, our findings are very similar to the previous study by Buckley and colleagues, whose study focuses on infants requiring cardiac surgery. They showed an overall CMA yield of 14% in their cohort, similar to our yield of 14.6% [18]. They also found a high proportion of abnormal cytogenetic testing in the septal class (33%) somewhat comparable to our high yield of this class.

The results from our study also identified additional CHD classes that were enriched for diagnostic CMA findings, primarily the conotruncal and LVOTO classes that had some of the highest CMA yields. While diagnostic findings were enriched in syndromic CHD patients in the conotruncal class, the LVOTO class had more diagnostic CMA results in patients with apparently isolated CHDs. Importantly, these were patients who were assessed as having a low likelihood of positive genetic testing on initial medical geneticist consultation who subsequently received a diagnosis based on CMA. In a majority of cases, the CNVs were identified in infants without ECAs or dysmorphic features.

While previous literature has recommended cytogenetic testing for specific CHD classes or for syndromic presentations [13,14], our results and experiences indicate that clinically significant CMA results are found across all CHD classes and in those with apparently isolated as well as syndromic presentations. These findings support wider adoption of CMA testing (or other testing that can reliably and consistently detect CNVs) for CHD cohorts independent of the CHD type or suspicion for syndromic presentations. Further research on the utility of genetic screening for CHD classes with low proportions of diagnostic and/or clinically meaningful results is needed.

### 4.2. Dysmorphic Features and/or ECAs Are Associated with Increased Diagnostic Yield

In our study, every inpatient neonate or infant with CHD had an evaluation by a geneticist. Dysmorphic features or ECAs were considered features of a possible genetic syndrome and were categorized as CHD + ECA (syndromic) to distinguish this group of patients from those with apparently isolated CHDs. We found a diagnostic yield for CMA of 26.5% in the former group and 6.5% in the latter. To our knowledge, this is the first study that has reported CMA yields in syndromic and apparently isolated CHDs in a comprehensive cohort in which all patients had evaluations for dysmorphology by a geneticist. At a previous institution, we reported yields of 21.7% for syndromic CHDs and 11% for isolated CHDs, but only a subset of patients had evaluation by a medical geneticist and therefore dysmorphic features were not included, potentially accounting for the differences in proportions between the two studies [39]. Another study, by Geddes et al., comprehensively evaluated and tested all inpatient infants with CHDs but did not specify extracardiac findings [20]. The testing yield for CNVs in that study was 22.6%, which is slightly higher than our overall yield of 14.6%. A study by Buckley et al. also included all inpatient CHD infants but did not routinely involve a geneticist evaluation and only performed cytogenetic testing in 51% of the cohort [18]. The study by Ahrens-Nicklas et al. was designed to specifically address the utility of genetic testing in the CHD population and provided diagnostic yields across testing modalities. A geneticist evaluated all patients in the study, but these were a subset of the overall infants with CHDs during the study time period and therefore likely include few cases with apparently isolated CHDs. Nevertheless, they found that ECA was not an independent predictor of positive testing results, but dysmorphic features identified by a geneticist resulted in a 7-fold increased likelihood of a diagnosis [37]. The study by Shikany et al. similarly found an increased association of dysmorphic features with abnormal genetic findings (odds ratio 3.5) and that infants who lacked dysmorphic features had similar frequencies of abnormal genetic testing results whether CHD was isolated or associated with ECA [39]. These studies suggest that dysmorphology may be more important, or at least of equal significance, to other congenital anomalies with regard to a priori probability of diagnostic genetic testing.

### 4.3. Comprehensive Assessment of Infants with CHDs Identifies Patients in Whom a Genetic Abnormality Was Not Suspected

With the initial emergence of CMA as a diagnostic tool for patients with CHDs and syndromic presentations, groups investigated the use of CMA in patients with apparently isolated CHDs and identified 3.8–4.3% yield [35,36], concluding that while lower than in patients with a syndromic presentation, the findings resulted in changes in clinical management and thus demonstrated clinical utility. Nevertheless, the field has been slow to adopt broad-based testing postnatally although prenatal guidelines for CMA in fetuses with severe CHD exist. The current study allows us to begin to address the problem of the missing denominator in previous studies by comprehensively evaluating all CHD infants within the intensive care unit for dysmorphic features and other evidence of a genetic syndrome. The results indicate that 6.5% of the CHD infants with diagnostic CMA findings did not have features suggestive of a genetic syndrome on exam by a geneticist.

Genetic conditions such as 8p23.1 deletion/duplication, 15q11.2 deletion, 16p11.2 duplication, 16p13.3 deletion/duplication, and 22q11.2 duplication may not be associated with dysmorphisms particularly in neonates. These conditions may have variable penetrance of neurodevelopmental abnormalities and other medical problems. Early identification of these disorders allows for proactive medical management and developmental intervention and surveillance in order to improve long-term outcomes. In addition, these CNVs are often inherited from a parent, and recurrence risk counseling is important. Finally, in some cases, a parent who carries a CNV may themselves have medical problems or intellectual disability related to their previously undiagnosed genetic finding, which may be important for providers caring for their child. Some classes (i.e., APVR and heterotaxy) did not have CMA results that explained the cardiac phenotypes but had clinically significant CMA incidental findings important for patient management and genetic counseling.

Notably, CHDs have been reported in up to 9% of people with the BP1-BP2 15q11.2 deletion, and we identified a few cases in this cohort [23,24]. Up to 0.5–1.0% of the general population may have these variants, and the associated phenotype of 15q11.2 copy-number variants is variable. The phenotype can include neurodevelopmental and behavioral differences, though some people are apparently unaffected [40]. However, given the variable clinical presentations and incomplete penetrance of these 15q11.2 CNVs and the general population prevalence of CHDs, further research on this CNV and its possible causal association for CHDs is necessary.

One particularly interesting finding of this study is that the LVOTO class of CHD had a high proportion of these CNVs associated with incomplete penetrance of CHD and neurodevelopmental differences. Additional studies are needed to better define the prevalence of these more recently identified CNVs in specific CHD classes and to further identify factors that increase the penetrance of CHD in conjunction with these CNVs. In addition, it is of great importance for longitudinal follow-up of intellectual outcome and other medical concerns. The findings from our study, in combination with previous work, show that CMA provides benefit for patients with CHDs not presenting in an overtly syndromic fashion.

### 4.4. Incorporation of Evaluation by Medical Geneticist Increases Syndrome Diagnosis and Molecular Genetic Testing is Additive

Of 376 patients with first-tier CMA in our cohort, 75 had additional molecular testing as inpatients given high suspicion for a genetic diagnosis with 9 (12%) having diagnostic results. The actual number of patients with an identifiable monogenic cause of their isolated CHD or a genetic syndromic cause will require longitudinal follow-up of the cohort with ongoing assessment of dysmorphology and neurodevelopment, as well as additional genetic testing in select cases. However, within the limitations of the inpatient evaluation, which included only one or two assessments by a geneticist, nonexhaustive genetic testing, and critically ill status of the infant, the sensitivity of the genetics evaluation and testing for detection of genetic abnormalities was 68.8% and the specificity was 78.8%. The sensitivity, as discussed above, was reduced by the number of nondysmorphic infants with isolated CHD who had diagnostic CMA results.

Molecular genetic testing performed as a second-tier test after negative CMA in patients with high suspicion for a genetic syndrome increased both the sensitivity and specificity by reducing the number of false-positive syndromic cases and increasing the true-positive number. It is likely that additional longitudinal follow-up of this cohort will lead to additional diagnoses. Table 6 documents the proportion of diagnostic molecular findings from other studies. In cases ascertained prenatally with severe CHD as part of the Netherlands registry, exome testing was done sequentially in patients with CHD + ECA with a yield of 5.8% leading the authors to conclude that if timing allows prenatally, exome testing should be offered after negative CMA. They emphasize that the involvement of a geneticist is critical [28]. The other studies of postnatal cohorts all reported higher yields than the 12% found in our study, with rates of 17% [37], 27% [39], and 36% [20]. In all of these studies, molecular testing was sent as second tier based on a geneticist’s evaluation. Given the date ranges of some of the studies, exome testing was ordered less frequently than molecular panels. Differences in the yields of testing are at least partially related to the increasing number of panels available over time.

When considering those diagnoses that were confirmed via molecular genetic testing in addition to CMA, the overall diagnostic proportion in our study increased to approximately 17%. These molecular genetic diagnoses would not have been identified using CMA alone, and testing would likely not have been ordered or completed without formal genetics consultation. The use of molecular genetic testing strategies beyond CMA was dependent on patient presentation and clinical context and appeared to be provider-specific. Standardized use of ES/WGS for pediatric CHD cohorts should be explored, including whether there are significant improvements in diagnostic yield above the current standard. It will be critical as our field moves to the adoption of clinical ES/WGS as a first-tier test in infants with CHDs that genetic testing laboratories provide assurance of consistent and reliable CNV coverage prior to supplanting CMA. Some ES/WGS studies have variably reported on CNVs in their cohorts, and ES specifically has current limitations with consistent CNV detection [41]. Recent studies using ES/WGS indicate diagnostic or clinically actionable findings in up to 20–30% of cases [4,7]; however, other work has questioned whether strategies for broad ES demonstrate improvement versus the standard case-by-case ordering by medical genetics providers [42], indicating the need for further investigation.

About half of the abnormal CMAs and a significant proportion of sequencing-based tests in our study resulted in VUSs, and while evidence of pathogenicity is currently absent or limited, it is possible some could be contributing to CHD development. Teams implementing genetic testing for pediatric CHD populations should involve experts in genetics for best practices with results interpretation and investigation of VUSs. Large CHD patient registries with reported genetic testing findings will also be critical for improving variant interpretation for such findings [43]. Therefore, this and similar studies are important for further research into the genetic etiologies of CHDs.

### 4.5. Redundant Genetic Testing Occurs Frequently without a Clinical Algorithm

Clinically significant CNVs were found in nearly 15% of CHD patients in this cohort where CMA was completed, and there were few clear cases where other cytogenetic testing would have been sufficient for diagnosis. In general, the clinical algorithm was very well implemented. Some of the CHD infants who were not tested can be attributed to logistical issues related to test ordering and the realities of inpatient intensive care unit workflows. In addition to the current study, there have been two recent studies in infants with critical CHDs utilizing a comprehensive clinical algorithm for genetic testing with CMA [20,39]. In the study by Geddes et al., 98% of inpatient CHD infants underwent evaluation by a geneticist and genetic testing [20], whereas in the study by Skikany et al. [39] and the current study, approximately 85–90% of patients underwent genetic testing. In contrast, in studies where a clinical algorithm is not in place, there is documentation of undertesting or redundant testing [16,18,20,38] leading to cost inefficiencies and missed diagnoses. Standardized genetic testing strategies and clinical algorithms that reduce practice variation have been shown to be cost-effective for CHD patients [38] while also increasing the number of actionable diagnoses.

### 4.6. Limitations

This study did not incorporate clinical diagnoses based on exam and should be considered a minimum estimate of genetic diagnoses in infant CHD populations. Additionally, our inpatient program was not uniformly consulted on all common aneuploidies (especially if prenatally diagnosed). If including common aneuploidies where only FISH or karyotype was completed, there were an additional 13 diagnostic cases raising the overall cohort genetic testing yield to 78/440 (17.7%). Similarly, the use of non-CMA genetic testing was variable and not standardized, so it is difficult to extrapolate diagnostic yields if molecular genetic testing (including exome-based strategies) had been utilized more consistently. This study only assessed genetic testing completed in the inpatient setting in neonates/infants, and syndrome recognition can be challenging in this population. As this cohort is followed on an outpatient basis, further information will be obtained with additional phenotypic assessments and genetic testing, if indicated. While other tests were rarely used compared to CMA, some of them confirmed diagnoses in a limited number of cases in the inpatient cohort. Therefore, the 14.6% yield of CMA should not be viewed as a proxy for the overall proportion of all possible genetic diagnoses in CHD patients. Comprehensive studies assessing cytogenetic and molecular genetic testing are necessary to fully explore total genetic diagnoses in CHD cohorts. Last, this was a pediatric (neonatal and infantile) cohort, so it is unclear if these findings have external validity to other CHD populations, specifically adult CHD patients and CHD patients not seen in the cardiac intensive care setting.

## 5. Conclusions

Cumulative evidence indicates that genetic testing is important in infants with critical CHDs requiring hospitalization in the first year of life. Notably, this occurs across all CHD classes, providing a rationale for widespread use. Although diagnostic yield is higher in CHD infants suspected to have a genetic syndrome, even careful dysmorphology exams by geneticists are not completely sensitive to all diagnoses at this age such that 6.5% of patients with apparently isolated CHDs have diagnostic findings by CMA, again arguing for widespread use of testing. Geneticists’ evaluations are particularly important for second-tier molecular testing in infants. These tests are additive with diagnostic rates ranging from 6 to 36% depending on the cohort. Dysmorphic features are strongly correlated with diagnostic testing results across modalities. Incorporation of a clinical algorithm for genetic evaluation and testing is important to decrease the number of patients with missed diagnoses and to decrease redundant testing and cost inefficiencies. Results to date indicate that these clinical tests change management for patients with a new genetic diagnosis and are impactful for families. Future studies are required to determine long-term impact on outcomes. There is reason for optimism for improving the yield of genetic testing for CHDs even further with further refinement of variant interpretation and the ability to utilize ES/WGS. However, a note of caution is also indicated. It has been more than 15 years since the introduction of CMA testing, and its use in infants with CHDs is still highly provider- and institution-dependent with few sites adopting comprehensive approaches despite its impact on care and its relatively low cost as a proportion of the overall resource utilization of infants with severe CHDs. As the genetic testing target continues to move and ES/WGS are now the new landmarks, well-designed comprehensive, standardized clinical studies that address utility are imperative to facilitate the implementation of best practices in this patient population where early genetic diagnoses are easily missed.

## Figures and Tables

**Table 1:** Phenotypic classification of neonates and infants with CHDs in the intensive care unit. August 2014–December 2019.

CHD Class	Total Number in Study Population (%)	Completed CMA Proportion	Number (%) Syndromic (ECA-Positive)	Number (%) Apparently Isolated (ECA-Negative)
APVR	14 (3.2)	92.9%	1 (7.1)	13 (92.9)
AVSD	13 (3.0)	76.9%	2 (15.4)	11 (84.6)
Complex	66 (15.0)	83.3%	5 (7.6)	61 (92.4)
Conotruncal	130 (29.6)	88.5%	46 (35.4)	84 (64.6)
Heterotaxy	35 (8.0)	85.7%	32 (91.4)	3 (8.6)
LVOTO	121 (27.5)	83.5%	24 (19.8)	97 (80.2)
RVOTO	37 (8.4)	86.5%	7 (18.9)	30 (81.1)
Septal	24 (5.4)	83.3%	15 (62.5)	9 (37.5)
Total	440 (100)	Average = 85.1%	132 (30.0)	308 (70.0)

**Table 2:** Proportion of chromosomal microarray abnormalities in CHD classes.

CHD Class	Proportion of Abnormal CMA Results #	Proportion of Clinically Significant Abnormal CMA Results #
APVR	23.1% (3/13)	7.7% (1/13)
AVSD	50.0% (5/10)	20.0% (2/10)
Complex	29.0% (16/55)	10.9% (6/55)
Conotruncal	28.7% (33/115)	19.1% (22/115)
Heterotaxy	20.0% (6/30)	3.3% (2/30) *
LVOTO	29.7% (30/101)	15.8% (16/101)
RVOTO	34.4% (11/32)	9.4% (3/32)
Septal	40.0% (8/20)	20.0% (4/20)
Total	N = 112	N = 55 *

# These proportions reflect the 376/440 patients in total that had CMA completed and not aneuploidy diagnoses made using non-CMA testing. * One of these cases is not counted towards the final overall diagnostic proportion. See text for details. APVR, anomalous pulmonary venous return; AVSD, atrioventricular septal defect; LVOTO, left ventricular outflow tract obstructive defect; RVOTO, right ventricular outflow tract obstructive defect.

**Table 3:** Diagnostic yield of chromosomal microarray by Botto class, stratified by extracardiac anomaly status.

CHD Presentation						
Syndromic (ECA-Positive)				Apparently Isolated (ECA-Negative)		
Botto Class (Level 3)	Counts (n)	Diagnostic CMA Results (n)	Proportion Clinically Significant CMA for Class	Counts (n)	Diagnostic CMA Results (n)	Proportion Clinically Significant CMA for Class
APVR	1	0	0.0% (0/1)	13	1	7.7% (1/13)
AVSD	2	2	100.0% (2/2)	11	0	0.0% (0/11)
Complex	5	4	80.0% (4/5)	61	2	3.3% (2/61)
Conotruncal	46	19	41.3% (19/46)	84	3	3.6% (3/84)
Heterotaxy	32	0	0.0% (0/32)	3	1	33.3% (1/3)
LVOTO	24	6	25.0% (6/24)	97	10	10.3% (10/97)
RVOTO	7	0	0.0% (0/7)	30	3	10.0% (3/30)
Septal	15	4	26.7% (4/15)	9	0	0.0% (0/9)
Total (n)	132	35	Overall: 26.5% (35/132)	308	20	Overall: 6.5% (20/308)

**Table 4:** Geneticists’ a priori assessment of the likelihood of genetic diagnosis.

CHD Class	Number of Clinically Significant CMAs Per CHD Class	Assessed as Low Likelihood of Genetic Abnormality	Assessed as High Likelihood of Genetic Abnormality
APVR	1	1 15q11.2 deletion (BP1-BP2)	0
AVSD	2	0	2 10 Mb duplication of 5p13.2-p11 Trisomy 21;
Complex	6	2 22q11.2 duplication; Xp22.31 deletion	4 2q22.1-q23.3 deletion (Mowat–Wilson syndrome, with 2% ROH and another Xq27.2 deletion); Recombinant chromosome 8 syndrome; Trisomy 18; 22q11.2 deletion
Conotruncal	22	3 22q11.2 deletion × 2; Xq28 deletion (*BRCC3*, familial moyamoya);	19 1p36 deletion syndrome; 39.31 Mb 3p22.2-pter duplication/1.68 Mb deletion of 12q24.33-qter; 6p23.2-p25.1 deletion/9q34 duplication; Trisomy 13; 16p11.2 deletion × 2; 20p12 deletion (Alagille); 22q11.2 deletion/21q22.3 duplication (5 Mb); 22q11.2 deletion/24% ROH; 22q11.2 deletion × 10
Heterotaxy	1	2 * 16p13.11 duplication (*MYH11*); 11p15.4 deletion (*HBB* globin cluster-carrier for β-thalassemia) *	0
LVOTO	16	10 7q11.23 duplication; 8p23.1 duplication × 2; 15q11.2 deletion (BP1-BP2) × 2; 15q11.2 deletion (BP1-BP2)/1p12 duplication (*NOTCH2*, Alagille syndrome); 16p11.2 duplication; 17p12 deletion (*PMP22*, risk for neuropathy); 22q11.2 duplication; Mosaic Turner syndrome	6 7q11.23 deletion (Williams syndrome); Trisomy 13; mosaic trisomy 13; 15q24.2-q24.3 duplication (2.2 Mb); 16p11.2 deletion × 2
RVOTO	3	3 16p11.2 duplication; 8p23.1 deletion; Tetrasomy X	0
Septal	4	0	4 2q22.1-q22.3 deletion (Mowat–Wilson syndrome); Trisomy 18; 22q11.2 deletion × 2

* This case was not included in the overall diagnostic proportion since it represents a secondary finding. APVR, anomalous pulmonary venous return; AVSD, atrioventricular septal defect; LVOTO, left ventricular outflow tract obstructive defect; RVOTO, right ventricular outflow tract obstructive defect.

**Table 5:** Molecular testing results.

Molecular Test	Tests (N)	Proportion of Diagnostic Results	Diagnostic Findings
Heterotaxy panel	20	5.0% (1/20)	*DNAH11*-related primary ciliary dyskinesia/heterotaxy confirmed with nasal ciliary biopsy
Exome	18	5.6% (1/18)	*IFT172*-related disorder/Joubert syndrome
Noonan/RASopathy panel	16	25% (4/16)	*PTPN11* × 2, *RAF1*, *HRAS*
Congenital anomalies CHD panel	26	0% (0/26)	N/A
*CHD7* (CHARGE syndrome)	4	5.0% (2/4)	*CHD7*
Aortopathy panel	2	0% (0/2)	N/A
Beckwith–Wiedemann syndrome/Russell–Silver syndrome methylation analysis	2	0% (0/2)	N/A
*ELN* (nonsyndromic supravalvular aortic stenosis)	2	0% (0/2)	N/A
Primary ciliary dyskinesia panel	2	0% (0/2)	N/A
Cardiomyopathy panel	1	0% (0/1)	N/A
CHD gene panel	1	0% (0/1)	N/A
Ciliopathies panel	1	0% (0/1)	N/A
Craniosynostosis panel	1	0% (0/1)	N/A
Exome + mtDNA panel	1	100% (1/1)	*MTTL1*
Gonadal dysgenesis panel	1	0% (0/1)	N/A
*JAG1* (Alagille)	1	100% (1/1)	*JAG1*
Limb reduction anomalies panel	1	0% (0/1)	N/A
*SLC2A1* (Glut-1 deficiency)	1	0% (0/1)	N/A
Total	101	10	

**Table 6:** Summary data from key publications on genetic testing in fetuses or infants with CHDs.

Study and Dates	Size	Source	Overall Testing Yield	Chrom or FISHYield	CNV Yield	VUS	Molec Yield	CMA Testing Yield ECA vs. iCHD	Key Findings
Prenatal									
[28] 2011–2016	217	Fetal echo database; CMA in 217/336	36.9%	29.5%	7.4%	7.4%	N/A	ECA 64.5% iCHD 22%	Type of CHD and presence of ECA impact yield
[29] 2012–2016	919 Pre = 542 Post = 185 No testing = 192	NL PRECOR registry; severe CHD with pre- or postnatal CMA	30.6%	23%	9.9% (4.2% 22q11.2)	2.7% CMA; 2.8% molec	5.8%	Prenatal ECA 28.7% iCHD 11.6%	Exome seq should be offered for CHD + ECA 2nd tier if time allows
[30] 2015	239	Cytogenetic labs; all fetuses with iCHD in France with CMA; TGA, htx, abn karyotype excluded	7.9% (CMA)	N/A	7.9%	2.5%	N/A	Only iCHD	3.1% ↑ yield even when 22q11.2 excluded; fetuses with iCHD benefit from CMA
Postnatal									
[31] 2006–2013	422 ECA = 260 iCHD= 162	CMA; reasons for testing, # not tested NR; median age 7	21.3%	12 cases (2.8%) found by CMA;	15.6% for P/LP 12.8% (P only)	NR	N/A	ECA 20.6% iCHD 9.3%	CMA as 1st-tier test; among syndromic, those with DD/ID or autism ↑ yield
[17,32,33]	208	Selected syndromic CHD with CMA	Range	N/A	6.6–20.7%	NR	N/A	ECA only	Useful testing in syndromic CHD cases without dx
[34]	40	CHD+ECA compared to iCHD, selected cohort of 20 each	12.5%	N/A	12.5%	NR	N/A	ECA 25% iCHD 0%	CMA identifies causes in CHD+ECA cases
[35,36]	151	CHD patients without syndromic features	Range	N/A	3.8–4.3%	NR	N/A	iCHD only	iCHD yield less than ECA but valuable
[16] 2008–2010	277 of whom 121 had CMA	All CHD infants with cytogenetic testing (277/1087 CHD)	15%	14%	3.2% cohort; 7% of CMA sub-group	22% CMA sub-group	N/A	ECA 12% iCHD 0%	Low proportion of CHD patients tested; high rate of redundant testing.
[18] 2010–2013	275 cytogenetic testing; 535 total	All infants with critical CHD	ND	22% (10% kayotype, 12% FISH)	14%	13%	N/A	NR	CMA yield highest in septal class; at least 18% redundant testing
[37] 2007–2012	364	CICU infants with genetics consultation only (total # CHD cases NR)	25% (9% prenatal, 16% post)	23% (of 182 chrom); 12% (of 147 FISH/MLPA)	9% (of 296 CMA)	8% (of 296 CMA)	17% (of 82 molec)	NR	CHD type influences yield; septal, AVC highest; dysmorphic features by geneticist = 7× ↑ likelihood of dx.
[38] Pre-protocol 2010–2014; post-protocol 2015–2016.	733 pre 158 post	STS database infants critical CHD; post-protocol all with genetics consultation	Pre: 26% Post: 36%	Pre: 18%; FISH 9%; Post: 76% FISH 26%	Pre: 24% Post: 22%	NR	NR	NR	Multiple testing ↓ post-protocol and testing rate ↑; rate of dx ↑; cost savings
[39] 2010–2015	293 213 iCHD, 80 ECA	All infants <1 month in CICU; subset had geneticist eval	26%	29.1% (23/79)	14.3% (30/210)	Included in CNV yield	5.8% of overall cohort; 27% of those tested (17/62)	ECA 21.7%* (13/60) iCHD 11% (17/150)	CHD class, specific ECAs, dysmorphic features associated with ↑ yield.
[20] 2015–2018	201	All infants critical CHD; excludes trisomies; all with eval by single geneticist	33%	17.8% (5/28) chrom 33.3% (9/27) FISH	22.6% (43/190)	2.1% (4/190)	35.7% (20/56)	NR	↑ dx rate, detection of complex phenotypes, incidental findings that alter management with inpatient cardiogenetics program; ↑ genetic testing utilization and ↓ redundant testing
This study 2014–2019	440 (376, with CMA completed)	All infants critical CHD; known chrom abn excluded; all with geneticist eval	18%	N/A	14.6% (55/376)	14.9% (56/376)	2.3% of overall cohort; 10.5% of those tested (10/95)	ECA 26.5% (35/132) iCHD 6.5% (20/308)	All CHD classes had P/LP CNVs; LVOTO often had CNVs in iCHD; conotruncal in ECA. Molecular testing additive

CHD, congenital heart defect; CICU, cardiac intensive care unit; CMA, chromosome microarray; CNV, copy number variant; ECA, extracardiac anomaly; FISH, fluorescence in situ hybridization; htx, heterotaxy; iCHD, isolated congenital heart defect; molec, molecular; N/A, not applicable; NL, Netherlands; NR, not reported; P/LP, pathogenic/likely pathogenic variants; TGA, transposition of the great arteries; VUS, variant of uncertain significance; * this study included VUS CMA findings as abnormal.

## Supplementary Materials

The following are available online at https://www.mdpi.com/article/10.3390/genes12081244/s1, Table S1: CMA findings and features in patients assessed at a low likelihood of abnormal CMA, Table S2: CMA findings and features in patients assessed at a high likelihood of abnormal CMA.
