# Supplementary material for: Genetic Evaluation of Inpatient Neonatal and Infantile Congenital Heart Defects: New Findings and Review of the Literature
Source: Genes (Basel). 2021 Aug 14;12(8):1244. doi: 10.3390/genes12081244 (PMC8391303; doi:10.3390/genes12081244)
Supplement: Supplementary file 1 [file genes-12-01244-s001.zip › genes-1265515-supplementary.pdf]

Supplemental Table 1. CMA findings and features in patients assessed at a low likelihood of abnormal CMA

CHD Class	Abnormal (n)	CMA Findings	Dysmorphic Features
APVR	1	15q11.2 deletion (BP1-BP2)	None
AVSD	0		
Complex	3	22q11.2 duplication	No features noted on exam
		22q11.2 deletion	No features noted on exam
		Xp22.31 deletion	No features noted on exam
Conotruncal	3	22q11.2 deletion	No features noted on exam, but increased chance of the disorder because of the conotruncal CHD
		22q11.2 deletion	No features noted on exam, but general concern for a syndrome because of the CHD plus imperforate anus
		Xq28 deletion (BRCC3, familial moyamoya)	Specific syndrome not noted on exam, but some minor anomalies (slightly low-set ears, bitemporal widening of the skull)
Heterotaxy	2	11p15.4 deletion (HBB globin cluster, carrier for beta-thalassemia)	No features noted on exam
		16p13.11 duplication (including MYH11)	No features noted on exam
LVOTO	10	7q11.23 duplication	Specific syndrome not noted on exam, but there was presence of: mild hypospadias, sacral dimple, and large first toes
		8p23.1 duplication	Non-dysmorphic
		8p23.1 duplication	Specific syndrome not noted on exam, but nevus flammeus on upper eyelids and nose
			No features noted on exam
		15q11.2 deletion (BP1-BP2) (x2)	No features noted on exam
		15q11.2 deletion (BP1-BP2)/1p12 duplication (of NOTCH2)	
		16p11.2 duplication	Specific syndrome not recognized, but case was noted to have mild frontal bossing
			No features noted on exam
		17p12 deletion (of PMP22)	No features noted on exam
		22q11.2 duplication	No features noted on exam
		Mosaic Turner syndrome	
RVOTO	3	8p23.1 deletion	Specific syndrome not noted on exam, but slight webbing of neck
		16p11.2 duplication	Specific syndrome not noted on exam, but case had slightly posteriorly rotated ears
		Hypertelorism, cranial molding, prominent occiput, overfolded ear helices	Abnormal appearing female genitalia with left-sided enlargement of the labia and unclear vaginal opening (?rectovaginal fistula)
Septal	0		

APVR, anomalous pulmonary venous return; AVSD, atrioventricular septal defect; LVOTO, left ventricular outflow tract obstructive defect; RVOTO, right ventricular outflow tract obstructive defect

Supplemental Table 2. CMA findings and features in patients assessed at a high likelihood of abnormal CMA

CHD Class	Abnormal (n)	CMA Findings	Dysmorphic Features	ECAs
APVR	0			
AVSD	2	10 Mb duplication 5p13.2-p11	Mildly macrocephalic, posteriorly-rotated and low-set ears, preauricular pit, large and slightly protruding tongue, redundant mucosal tissue on perianal skin, small umbilical hernia, small shallow sacral dimple, slight hypotonia possibly c/w sedation, weak suck	Macrocephaly
		Trisomy 21	Facial features c/w trisomy 21 (almond-shaped eyes, short nose, upslanting palpebral fissures	Duodenal atresia
Complex	3	Recombinant chromosome 8 syndrome	None	Communicating hydrocephalus, myelomeningocele
		22q11.2 deletion	Brachycephaly, reverse epicanthal folds, dysplastic ears, prominent nasolabial folds, mild laterally displaced nipples, mild hypotonia	
		2q22.1-q23.3 deletion (Mowat-Wilson syndrome)	Wide nasal bridge, short philtrum, ears with upturned/creased lobes, low-set ears, bridged palmar crease, clenched fists, mild-moderate abdominal distension	
Conotruncal	19	22q11.2 deletion (general features in aggregate)	Hooded upper eyelids, diminished supraorbital ridges, bulbous nose, short nasal bridge, cleft palate, micrognathia, retrognathia, hypospadias, ridging of metopic suture, brachycephaly, mild excessive nuchal skin, small palpebral fissures, downslanting palpebral fissures, cupped ear(s) with overfolded helices, ear anomalies (crumpled appearance, overfolding) posteriorly-rotated ears, thin upper lip, prominent nasolabial folds, displaced nipples, hypotonia, long/thin fingers	Hypotonia, hypocalcemia, micrognathia, retrognathia
		22q11.2 deletion/21q22.3 duplication (5Mb)	Small palpebral fissures, small/underdeveloped nasal bridge, short sternum, overlapping digits	IUGR, small bowel obstruction
		6p23.2-p25.1 deletion/9q34 duplication	Long tapered digits, transverse palmar creases	
		16p11.2 deletion (general features in aggregate)	Upslanting palpebral fissures, flattened nasal bridge, posteriorly-rotated ears	Butterfly vertebrae, hemivertebrae, cystic kidney, unilateral renal agenesis, rib anomalies
		1p36 deletion	Intrauterine growth restriction, low posterior hairline, eyes slightly upslanting, eyes deep-set, mildly hypoplastic nasal root, prominent lateral nasal pillars, bulbous nose with hypoplastic alae nasi, long philtrum, mildly thin upper lip, very ridged and highly arched palate, small chin, ears mildly low set and posteriorly-rotated, thickened helices, excess nuchal skin, right single palmar crease, somewhat spatulated distal fingertips with hypoplastic nails also seen in her toes, mildly long fingers and toes	
		Trisomy 13	Midface hypoplasia	Microphthalmia, bilateral postaxial polydactyly, right-sided congenital diaphragmatic hernia

		20p12 deletion (Alagille syndrome)	Slight facial asymmetry, slightly shortened right palpebral fissure, continuous horizontal palmar crease of the left hand	Microcephaly, posterior embryotoxon (consistent with Alagille syndrome)
		39.31Mb 3p22.2-pter duplication/1.68 Mb deletion of 12q24.33-qter		Micropenis, bilateral cryptorchidism, micro-retrognathia
Heterotaxy	0	None	No notable dysmorphic features	Most had heterotaxy or laterality-spectrum malformations (e.g., situs anomalies)
LVOTO	6	7q11.23 deletion (Williams syndrome)	Broad forehead, bitemporal narrowing, periorbital fullness, epicanthal folds, mildly cupped ears, wide mouth, broad nasal tip	Microcephaly, small testes, right inguinal hernia
		15q24.2-q24.3 duplication (2.2 Mb)	Retrognathia, mildly recessed nasal bridge, first toes with fetal fat pads	
		16p11.2 deletion (x2)		Butterfly vertebrae, hemivertebrae, cystic kidney, unilateral renal agenesis, rib anomalies
		Mosaic trisomy 13	Capillary hemangiomas, overfolded ear helices, mildly short palpebral fissures, epicanthal folds, prominent nasal root and nasal bridge, hypoplastic alae nasi, mild micrognathia	Non-osseous postaxial polydactyly
		Trisomy 13	Dysmorphic features (noted generally)	
RVOTO	0	None		
Septal	4	2q22.2-q22.3 deletion (including the ZEB2 gene c/w Mowat-Wilson syndrome)	Wide nasal bridge, short philtrum, ears with upturned/creased lobes BL, low-set ears, bridged palmar crease, clenched fists, mild-moderate abdominal distension	
		22q11.2 deletion	Subtle strabismus, small palpebral fissures, left ear with overfolded helix, low-set and cupped ears, thin upper lip	
		22q11.2 deletion	Mild brachycephaly, mildly flat and asymmetrical occiput, downslanting palpebral fissures, prominent superior helices, posteriorly-rotated ears, short nasal bridge, long and thin fingers, 2-3 toe overlapping, possible 5th toe camptodactyly	Mild generalized hypotonia
		Trisomy 18	Exam difficult due to intubation, facial features c/w trisomy 18	Small for age (IUGR), left diaphragmatic hernia, pontocerebellar hypoplasia

APVR, anomalous pulmonary venous return; AVSD, atrioventricular septal defect; LVOTO, left ventricular outflow tract obstructive defect; RVOTO, right ventricular outflow tract obstructive defect

Supplemental Table 3a. Descriptive statistics for conotruncal subtype lesions

Conotruncal Subtype Group	Conotruncal Subtype	Number (n)	Proportion (%)
Complex/Single Ventricle	DILV	1	0.77%
DORV	DORV	10	7.69%
	DORV w/ LVOTO	1	0.77%
	DORV w/ IAA (NOS)	1	0.77%
IAA	IAA (NOS)	8	6.15%
	IAA w/ LVOTO	1	0.77%
TA	TA	11	8.46%
	TA w/ IAA (NOS)	1	0.77%
	TA w/ LVOTO	1	0.77%
D-TGA	D-TGA	41	31.54%
	D-TGA/DORV	9	6.92%
	D-TGA/DORV w/ IAA (NOS)	1	0.77%
	D-TGA w/ IAA (NOS)	1	0.77%
	D-TGA w/ LVOTO	2	1.54%
TOF	TOF	30	23.08%
	TOF w/ PA	9	6.92%
	TOF w/ DORV	1	0.77%
	TOF w/ LVOTO	1	0.77%
**Total**		**130**	**100%**

DILV = double-inlet left ventricle, DORV = double-outlet right ventricle, D-TGA = dextro-transposition of the great arteries, IAA (NOS) = interrupted aortic arch (type not otherwise specified), LVOTO = left ventricular outflow tract obstruction (accompanying the primary conotruncal defect), PA = pulmonary atresia, TOF = tetralogy of Fallot

Supplemental Table 3b. Conotruncal subtype lesions represented in patients with cytogenetic diagnoses

**Syndrome/Cytogenetic Disorder**	**Conotruncal Subtype Lesions**
22q11.2 deletion	DORV, IAA (NOS), IAA w/ LVOTO, TA, TOF, TOF w/ PA
22q11.2 deletion/21q22.3 duplication (5 Mb)	DORV
6p23.3-p25.1 deletion/9q34 duplication	TOF
16p11.2 deletion	D-TGA, TOF
1p36 deletion	TOF
Trisomy 13	DORV w/ LVOTO
20p12 deletion (Alagille syndrome)	TOF
39.31 Mb 3p22.2-pter duplication/1.68 Mb deletion of 12q24.33-qter	DORV
Xq28 deletion (BRCC3, familial moyamoya)	TOF

DILV = double-inlet left ventricle, DORV = double-outlet right ventricle, D-TGA = dextro-transposition of the great arteries, IAA (NOS) = interrupted aortic arch (type not otherwise specified), LVOTO = left ventricular outflow tract obstruction (accompanying the primary conotruncal defect), PA = pulmonary atresia, TOF = tetralogy of Fallot
